# Physician and hospital determinants of levothyroxine treatment adequacy in low-risk papillary thyroid carcinoma

**DOI:** 10.3389/fendo.2026.1820537

**Published:** 2026-08-04

**Authors:** Juan J. Díez, Emma Anda, Victoria Alcazar, Juan C. Galofré, Cristina Familiar, María J. Pamplona-Civera, Silvia González-Martínez, Ana Herrero-Ruiz, María R. Alhambra, Alejandra Planas, Cecilia Sánchez-Ragnarsson, Tomás Martín, Beatriz Rodriguez-Jiménez, Mireia Mora, Aida Orois, Reinaldo Sánchez-Barrera, Julia Sastre

**Affiliations:** 1 Department of Endocrinology, Hospital Universitario Puerta de Hierro Majadahonda, Majadahonda, Spain; 2 Instituto de Investigación Sanitaria Puerta de Hierro Segovia de Arana, Majadahonda, Spain; 3 Department of Medicine, Universidad Autónoma de Madrid, Madrid, Spain; 4 Department of Endocrinology, Hospital Universitario de Navarra, Pamplona, Spain; 5 Department of Endocrinology, Hospital Severo Ochoa, Leganés, Spain; 6 Department of Endocrinology, Clínica Universidad de Navarra, Pamplona, Spain; 7 Department of Endocrinology, Hospital Clínico San Carlos, Madrid, Spain; 8 Department of Endocrinology, Hospital Royo Villanova, Zaragoza, Spain; 9 Department of Endocrinology. Hospital Universitario de Cabueñes, Gijón, Spain; 10 Instituto de Investigación Sanitaria del Principado de Asturias (ISPA), Oviedo, Spain; 11 Department of Endocrinology, Hospital Universitario de Salamanca, Salamanca, Spain; 12 Unidad de Gestión Clínica (UGC) Endocrinología y Nutrición, Hospital Universitario Reina Sofía, Córdoba, Spain; 13 Instituto Maimónides de Investigación Biomédica de Córdoba (IMIBIC), Córdoba, Spain; 14 Department of Endocrinology, Vall d’Hebron Hospital Campus, Barcelona, Spain; 15 Department of Endocrinology, Hospital Universitario Central de Asturias, Oviedo, Spain; 16 Department of Endocrinology, Hospital Universitario Virgen Macarena, Sevilla, Spain; 17 Department of Endocrinology, Hospital Clínic, Barcelona, Spain; 18 Department of Endocrinology, Hospital Universitario de Bellvitge, Barcelona, Spain; 19 Department of Endocrinology, Hospital Universitario de Toledo, Toledo, Spain

**Keywords:** ATA guidelines, differentiated thyroid cancer, dynamic risk stratification, levothyroxine therapy, physician and hospital determinants, thyrotropin suppression, treatment adequacy

## Abstract

**Background:**

For low-risk differentiated thyroid carcinoma (DTC) survivors with excellent or indeterminate response, the 2025 American Thyroid Association (ATA) guidelines endorse assay-specific reference-interval thyrotropin (TSH) targets, potentially simplifying levothyroxine titration versus ATA 2015 fixed cutoffs.

**Objective:**

To assess whether endocrinologist and hospital-level characteristics influence levothyroxine treatment adequacy in low-risk DTC with excellent/indeterminate response, and to compare adequacy under ATA 2015 versus ATA 2025 frameworks.

**Methods:**

Multicenter, retrospective study including 978 adults managed by 16 endocrinologists. Dynamic risk stratification (DRS) status and serum TSH at last visit defined adequacy: ATA 2015 (0.5–2.0 mcU/ml for excellent; 0.1–0.5 mcU/ml for indeterminate) vs ATA 2025 (within local reference interval for both). Physician and hospital characteristics were compared across adequacy strata. Multilevel logistic regression (random intercept for physician) evaluated patient- (age, follow-up, dose instability) and physician-level predictors.

**Results:**

Under ATA 2015 criteria, 383 of 954 (40.1%) were adequately controlled, compared with 650 of 963 (67.5%) under ATA 2025 criteria. In those with >2 years’ follow-up, adequacy increased from 41.5% (364/878) to 68.8% (608/884) from ATA 2015 to ATA 2025. Dose instability independently predicted lower adequacy in both frameworks (ATA 2015: aOR 0.183, 95% CI 0.129–0.260; ATA 2025: aOR 0.110, 0.078–0.156; both p<0.001). With ATA 2025, additional inverse associations included older age (aOR 0.980/year; p=0.001), female physician sex (vs male; aOR 0.244; p=0.037), and higher new-patient volume (aOR 0.967/patient; p=0.009).

**Conclusions:**

Adoption of ATA 2025 reference-range TSH targets substantially increases the proportion of low risk DTC survivors classified as adequately treated. Nevertheless, treatment adequacy remains strongly shaped by dose instability and selected provider workload attributes.

## Introduction

Differentiated thyroid cancer (DTC) is the most common endocrine tumor and is usually associated with an excellent prognosis, as most patients fall into the low-risk tumor group (papillary or follicular). Furthermore, most of these patients achieve an excellent or indeterminate response after initial treatment, resulting in very low recurrence rates (1, 2). Clinical decision-making has historically followed the 2015 American Thyroid Association (ATA) framework (3), which tied dynamic response categories (excellent, indeterminate, biochemical incomplete, structural incomplete) to fixed numeric thyrotropin (TSH) targets. In the 2025 ATA update (4), this paradigm shifts toward the DATA framework (diagnosis, risk/benefit assessment, treatment decisions, response assessment) and reference-interval–based TSH goals: for patients with excellent or indeterminate response, TSH within the laboratory-specific reference range is considered appropriate, whereas TSH below the lower limit of normal is recommended for biochemical or structural persistence. This change is intended to facilitate individualized care and streamline survivorship by avoiding unnecessarily narrow targets. In parallel, comparative method work indicates that modern TSH immunoassays show high analytical agreement and that prescribing practices—rather than assay variability—are the predominant source of deviation from guideline targets, reinforcing the practicality of the 2025 reference-based approach (5, 6).

The rationale for de-emphasizing routine TSH suppression in low-risk disease is strengthened by contemporary outcome data. A large multicenter cohort and a systematic review and meta-analysis found no clear survival advantage of sustained TSH suppression in intermediate/high-risk DTC, while documenting greater risks of skeletal and cardiovascular adverse events under long-term supraphysiologic therapy (7); these concerns apply even more saliently to low-risk survivors. Recent narrative and systematic reviews likewise highlight that the balance of benefit and harm often disfavors aggressive suppression in low-risk cohorts, supporting a move toward reference-range TSH targets for excellent/indeterminate responders (5, 6, 8).

Despite these advances, real-world attainment of guideline-concordant TSH targets remains poor and variable. In a multicenter study from Turkey involving 1,125 DTC patients, only 29.2% were at target; 50.4% were overtreated and 20.4% undertreated (5). A Thai center reported that among low-risk patients the proportion at target was 8.8% at 12 months and 19.6% at last follow-up, with years in practice ≥10 independently associated with severe suppression, suggesting practice inertia (6). A recent Spanish multicenter cohort focused on low-risk disease found levothyroxine adequacy of 26.0% at 12 months increasing to 39.5% at last visit; in this setting, dose instability was the main determinant of long-term inadequacy, whereas patients with an excellent response were more often over-suppressed (8). Together, these observations underscore that adequacy is not merely a patient-level phenomenon; it appears to vary with clinicians’ behaviors and potentially with organizational features of the hospitals in which care is delivered (9, 10). Moreover, analyses comparing platforms show that assay choice contributes little to clinical discordance, shifting attention toward prescription patterns and system-level implementation (11–13).

This is therefore a timely moment to reassess levothyroxine management. The clinical community is transitioning from ATA 2015 numeric targets to ATA 2025 reference-based goals, a change that should, in principle, simplify dose titration for excellent/indeterminate responders and mitigate the cardiovascular and skeletal risks associated with unnecessary suppression (4). Understanding whether clinician- and center-level characteristics shape the likelihood of achieving adequacy under these evolving standards is essential to inform targeted implementation strategies and quality improvement (14, 15). Hence, the objective of this study has been to examine whether the professional characteristics of endocrinologists, as well as hospital-level features, influence the adequacy of levothyroxine treatment in low-risk DTC patients who achieve an excellent or indeterminate response to therapy.

## Methods

### Study design

As detailed previously (8), this multicenter retrospective study included adult patients (older than 18 years) with histologically confirmed low-risk DTC, of which 91.4% were papillary and 8.6% were follicular carcinoma. Surgical treatment comprised lobectomy (8.0% of cases) or total/near-total thyroidectomy (92.0%), with or without postoperative radioiodine (RAI) ablation. Clinical, pathological, and biochemical data, as well as imaging findings during follow-up, were collected, and the information required for dynamic risk stratification (DRS) at the last available visit within the study period (January 1–December 31, 2024) was compiled. Among patients with more than two years of follow-up, levothyroxine dose stability (stable vs. unstable) was assessed. Levothyroxine dose stability was defined based on dose adjustments during the 12 months preceding the last follow-up visit. Patients were considered to have a stable dose if no changes in levothyroxine dose were recorded during this period, and unstable if one or more dose adjustments were required. This variable was intended to reflect variability in achieving or maintaining target TSH levels in routine clinical practice, rather than appropriateness of clinical decision-making.

At diagnosis, patients were classified using the tumor–node–metastasis (TNM) staging system in accordance with the 8th edition of the American Joint Committee on Cancer/Union for International Cancer Control (AJCC/UICC) criteria (16). All individuals met the criteria for low risk of recurrence as defined by the 2015 American Thyroid Association (ATA) guidelines (3). All data were collected as part of standard clinical care. The study was conducted in accordance with the Declaration of Helsinki (as revised in 2013). Patient confidentiality was ensured in compliance with national regulations, and the study protocol was approved by the local ethics committee of Hospital Universitario Puerta de Hierro Majadahonda (Madrid, Spain) (approval number PI 157/24).

### Patient selection

**Figure 1:**
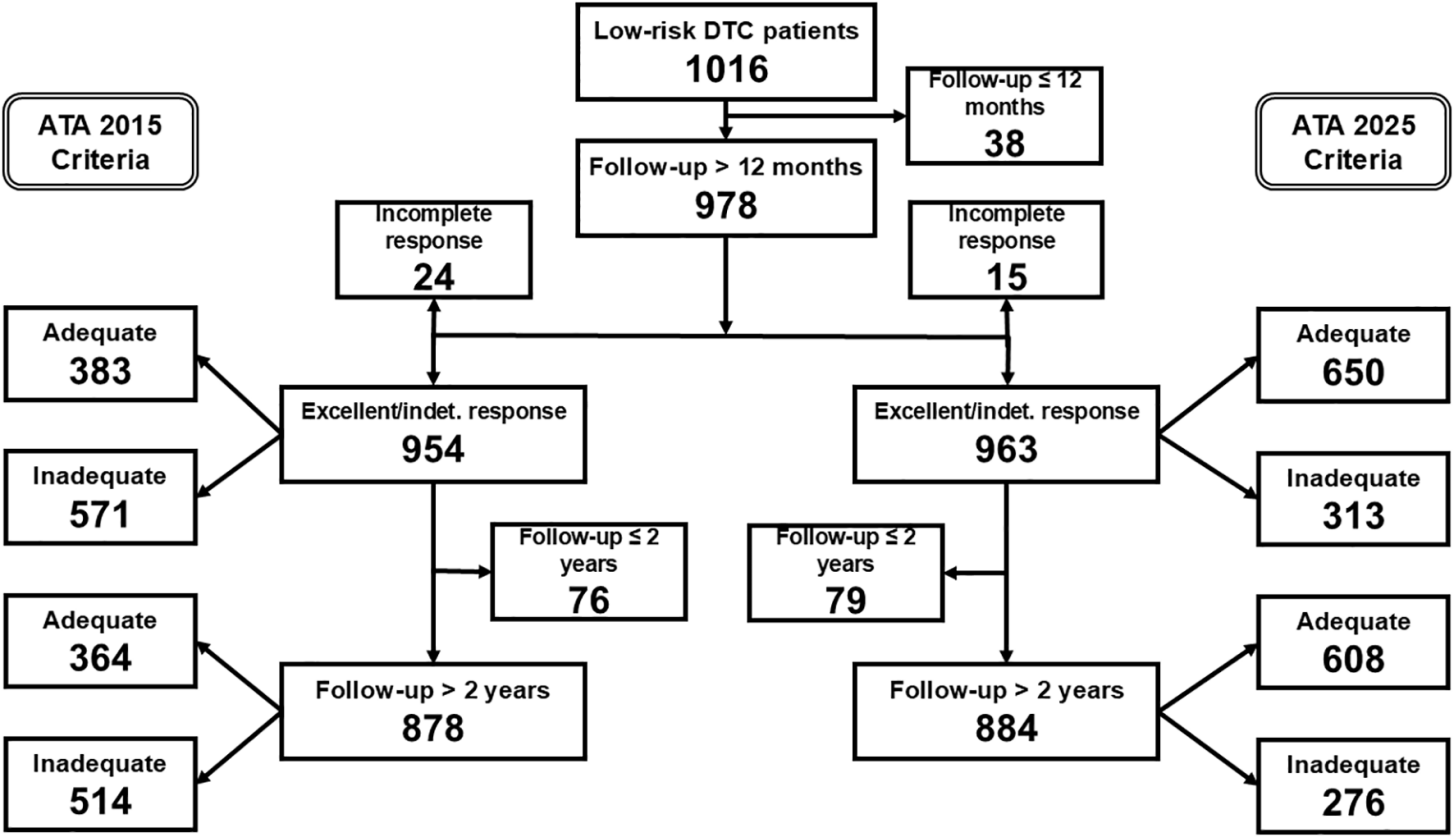
Flowchart of the study population and assessment of levothyroxine treatment adequacy. DTC, differentiated thyroid carcinoma; ATA, American Thyroid Association; indet., indeterminate.

For the purposes of this study, from the initial cohort of 1,016 low-risk patients we first selected 978 individuals with a disease duration >12 months (**Figure 1**). Applying the ATA 2015 DRS categories at end of follow-up in these 978 patients yielded the following responses: excellent in 761 (77.8%), indeterminate in 193 (19.7%), biochemical incomplete in 20 (2.0%), and structural incomplete in 4 (0.4%). For the primary analyses, we retained the 954 patients with excellent or indeterminate response; among them, 383 (40.1%) had adequate levothyroxine control and 571 (59.9%) were inadequately controlled. Because dose stability was only assessed in patients with >2 years of follow-up, a cohort of 878 such patients was used for the multilevel regression; among these, 364 (41.5%) were adequately controlled and 514 (58.5%) inadequately controlled.

Using the ATA 2025 criteria, 11 (1.1%) patients with biochemical incomplete response and 4 (0.4%) with structural incomplete response were excluded, leaving 963 patients with excellent (850, 86.9%) or indeterminate (113, 11.6%) response for the adequacy analysis. In this group, 650 (67.5%) were adequately controlled and 313 (32.5%) inadequately controlled. For the multilevel logistic regression, we selected patients with >2 years of follow-up, totaling 884 subjects, of whom 608 (68.8%) had adequate control and 276 (31.2%) inadequate control.

### Assessment of disease response status and treatment adequacy

DRS was evaluated according to the ATA 2015 and ATA 2025 guidelines, based on serum thyroglobulin, anti-thyroglobulin antibodies, and imaging studies obtained at the patients’ last visit (3, 4). Levothyroxine treatment adequacy was assessed using each patient’s serum TSH concentration at last follow-up visit. Patients were considered adequately controlled when their TSH fell within the therapeutic target recommended by the corresponding ATA guideline. Briefly, under ATA 2015, patients with an excellent response were considered adequately controlled when TSH was 0.5–2.0 mcU/ml whereas those with an indeterminate response were considered adequately controlled when TSH was 0.1–0.5 mcU/ml. In contrast, under ATA 2025, patients with either excellent or indeterminate response were deemed adequately controlled when their TSH was within the local reference interval of the participating hospital.

### Laboratory procedures

Serum TSH concentrations were measured using the standard procedures implemented at each participating hospital. Details of the TSH immunoassays used by participating centers, including manufacturers, analytical platforms, and laboratory-specific reference intervals, are provided in **Supplementary Table 1** (**Supplementary Material**). The lower limit of the TSH reference interval ranged from 0.25 to 0.55 μIU/mL, and the upper limit ranged from 4.2 to 5.3 μIU/mL. All participating clinical laboratories employed standardized assays and complied with established quality standards.

### Statistical analysis

Quantitative variables are expressed as median (interquartile range, IQR). Categorical variables are described using absolute values, ratios, or percentages. Proportions were compared using the chi-square test or Fisher’s exact test. Comparisons of quantitative variables between two groups were conducting using the Mann-Whitney *U*-test. We used two multilevel logistic regression models to account for the hierarchical structure of the data, with patients (level 1) nested within physicians (level 2). The dependent variables in these models were the adequacy of levothyroxine treatment using the ATA 2015 and ATA 2025 criteria. Patient-level predictors included age, follow-up duration, and dose instability, while physician-level predictors included gender, practice setting, hospital size, years in medical practice, thyroid case-mix, and volumes of new and follow-up patients. A generalized linear mixed model (GLMM) with a logit link and a random intercept for physician was fitted using the Laplace approximation. Degrees of freedom were estimated using the Satterthwaite method. Results are reported as adjusted odds ratios (aOR) with 95% confidence intervals, obtained by exponentiating coefficients on the logit scale. For interpretability, scaled effects were calculated (aOR per 5 years of age and per 25 patients for volume variables). All analyses were conducted in IBM SPSS Statistics v22. All statistical tests were two-sided and differences were considered significant when P <0.05.

## Results

### Participating endocrinologists and study patients

This study includes data from 16 endocrinologists and 978 patients. Key physician characteristics, together with the number of patients included in each category, are presented in **Table 1**. Most endocrinologists were women, worked exclusively in the public sector, were based in medium-to-large hospitals, and had substantial experience both in medical practice and in the management of thyroid disease.

**Table 1:** Main characteristics of the endocrinologists participating in the study, as well as the number of patients assigned to each of the physician categories considered.

	Endocrinologists (n=16)	Patients treated (n=978)
Patients’ characteristics		
Age, years		48 (38–59)
Time of follow-up, months		81 (48–144)
Dose stability*		
Stable		588 (60.1)
Unstable		310 (31.7)
Endocrinologists’ features		
Doctor’s gender		
Female	12 (75.0)	729 (74.5)
Male	4 (25.0)	249 (25.5)
Place of employment		
Public	13 (81.3)	821 (83.9)
Private	1 (6.3)	70 (7.2)
Public and private	2 (12.5)	87 (8.9)
Hospital size, beds		
251-500	4 (25.0)	260 (26.6)
501-1000	9 (56.3)	506 (51.7)
>1000	3 (18.8)	212 (21.7)
Time in medical practice, years	20 (15–30)	
Time in medical practice		
≤20 years		436 (44.6)
>20 years		542 (55.4)
Proportion of patients with thyroid disease		
<30%	1 (6.3)	57 (5.8)
31-60%	11 (68.8)	594 (60.7)
>60%	4 (25.0)	327 (33.4)
Annual volume of new patients	25 (15.8-37.5)	
Annual volume of new patients		
≤25		644 (65.8)
>25		334 (34.2)
Annual volume of patients in follow-up	215 (135–390)	
Annual volume of patients in follow-up		
≤215		391 (40.0)
>215		587 (60.0)

Data are the median (IQR) for quantitative variables, and the number (percentage) for categorical variables.

*Dose stability was evaluated in the 898 patients who had a follow-up period of more than 2 years.

Patients had a median age of 48 years [interquartile range (IQR), 38–59] and a median follow-up of 81 (48–144) months. The majority of patients were cared for by female physicians (74.5%), practicing exclusively in the public system (83.9%), and in hospitals with 251–1000 beds (78.3%). Nearly all patients (94.1%) were managed by physicians whose thyroid-disease workload exceeded 30% of their professional activity. A large proportion were followed by endocrinologists with >20 years of experience (55.4%) and with an annual follow-up caseload of >215 patients (60%).

### Comparison of adequately and inadequately controlled patients

**Table 2** compares physician characteristics among patients classified according to levothyroxine treatment adequacy using both the ATA 2015 and ATA 2025 criteria. When patients were grouped by adequacy under ATA 2015 criteria, levothyroxine adequacy was significantly associated with male physician sex, hospital size, years in practice, the proportion of thyroid-disease patients in the clinic, and the annual volume of new patients. In contrast, when adequacy was defined according to ATA 2025, significant associations were observed with hospital size, years in medical practice, the proportion of thyroid-disease patients, and annual patient volumes, including both new and follow-up visits (**Table 2**).

**Table 2:** Comparison of endocrinologists’ characteristics in patients with adequacy and inadequacy of levothyroxine treatment in the group of subjects with excellent or indeterminate response at the last follow-up visit according to the ATA 2015 and 2025 criteria.

	Adequacy of levothyroxine therapy in patients with excellent or indeterminate response					
	Evaluation using ATA 2015 criteria (n=954)			Evaluation using ATA 2025 criteria (n=963)		
	Adequate (n=383)	Inadequate (n=571)	P value	Adequate (n=650)	Inadequate (n=313)	P value
Doctor’s gender			0.023			0.693
Female	270 (70.5)	441 (77.2)		481 (74.0)	236 (75.4)	
Male	113 (29.5)	130 (22.8)		169 (26.0)	77 (24.6)	
Place of employment			0.652			0.852
Public	325 (84.9)	478 (83.7)		544 (83.7)	264 (84.3)	
Private	58 (15.1)	93 (16.3)		106 (16.3)	49 (15.7)	
Hospital size, beds			0.010			0.027
251-500	81 (21.1)	167 (29.2)		178 (27.4)	74 (23.6)	
501-1000	205 (53.5)	291 (51.0)		319 (49.1)	182 (58.1)	
>1000	97 (25.3)	113 (19.8)		153 (23.5)	57 (18.2)	
Years in medical practice	30 (16–35)	28 (16–30)	0.010	30 (16–30)	20 (15-30)	0.001
% patients with TD			<0.001			0.001
<30%	22 (5.7)	31 (5.4)		30 (4.6)	25 (8.0)	
31-60%	199 (52.0)	380 (65.5)		379 (58.3)	205 (65.5)	
>60%	162 (42.3)	160 (28.0)		241 (37.1)	83 (26.5)	
Annual patient volume, new	25 (20-40)	25 (15-40)	0.007	25 (20-40)	25 (10-30)	<0.001
Annual patient volume, FU	350 (150-400)	350 (130-400)	0.253	350 (150-400)	180 (130-380)	<0.001

Data are the median (IQR) for quantitative variables, and the number (percentage) for categorical variables. ATA, American Thyroid Association; TD, thyroid disease; FU, follow-up

Private includes physicians with private practice associated or not with public practice.

Finally, patient-level factors associated with adequacy differed by guideline set. Under ATA 2015, adequacy correlated with longer follow-up duration and dose stability. Under ATA 2025, adequacy correlated with older patient age, longer follow-up, and dose stability (**Supplementary Table 2**, **Supplementary Material**).

### Multilevel logistic regression analysis

The results of the multilevel logistic regression analysis using the GLMM with the adequacy of levothyroxine treatment as the explained variable are described in **Tables 3** and **4**.

**Table 3:** Adjusted odds ratios from multilevel logistic regression for adequacy of levothyroxine therapy in patients who achieved an excellent or indeterminate response at last visit according to ATA 2015 criteria.

Predictor	aOR	95% CI	P-value	Scaled effect	aOR (scaled)	95% CI (scaled)
Patient-level predictors						
Age, years	1.002	(0.991, 1.013)	0.660	Per 5 years	1.010	(0.956, 1.067)
Time of follow-up, months	1.000	(0.997, 1.002)	0.739	—		
Dose instability	0.183	(0.129, 0.260)	<0.001	—		
Physician-level predictors						
Physician sex, female	0.882	(0.204, 3.827)	0.862	—		
Public practice	0.751	(0.248, 2.282)	0.601	—		
Hospital size, ≤1000 beds	0.819	(0.232, 2.886)	0.746	—		
Years in practice	1.014	(0.958, 1.075)	0.606	—		
Thyroid case-mix, >60%	1.962	(0.294, 13.105)	0.470	—		
New patients volume	0.992	(0.965, 1.019)	0.533	Per 25 patients	0.819	(0.407, 1.608)
Follow-up patients volume	1.001	(0.999, 1.002)	0.642	Per 25 patients	1.025	(0.975, 1.051)

Values are aOR with 95% confidence intervals and p-values.

Patients (level-1) nested within physicians (level-2). Model: GLMM with random intercept for physician; binomial distribution and logit link. ATA, American Thyroid Association; aOR, adjusted odds ratio; CI, confidence interval; GLMM, generalized linear mixed model.

**Table 4:** Adjusted odds ratios from multilevel logistic regression for adequacy of levothyroxine therapy in patients who achieved an excellent or indeterminate response at last visit according to ATA 2025 criteria.

Predictor	aOR	95% CI	P-value	Scaled effect	aOR (scaled)	95% CI (scaled)
Patient-level predictors						
Age, years	0.980	(0.969, 0.992)	0.001	Per 5 years	0.905	(0.852, 0.961)
Time of follow-up, months	0.998	(0.996, 1.001)	0.227	—		
Dose instability	0.110	(0.078, 0.156)	<0.001	—		
Physician-level predictors						
Physician sex, female	0.244	(0.065, 0.915)	0.037	—		
Public practice	1.339	(0.502, 3.579)	0.545	—		
Hospital size, ≤1000 beds	1.280	(0.419, 3.904)	0.651	—		
Years in practice	0.962	(0.914, 1.013)	0.135	—		
Thyroid case-mix, >60%	1.127	(0.163, 4.826)	0.885	—		
New patients volume	0.967	(0.943, 0.991)	0.009	Per 25 patients	0.427	(0.229, 0.799)
Follow-up patients volume	1.000	(0.999, 1.002)	0.623	Per 25 patients	1.000	(0.975, 1.051)

Values are aOR with 95% confidence intervals and p-values.

Patients (level-1) nested within physicians (level-2). Model: GLMM with random intercept for physician; binomial distribution and logit link. ATA, American Thyroid Association; aOR, adjusted odds ratio; CI, confidence interval; GLMM, generalized linear mixed model.

Using ATA 2015 criteria in 878 patients (16 physicians), only dose instability showed an independent association with adequacy, indicating lower odds of adequate treatment (aOR = 0.183, 95% CI 0.129–0.260; p < 0.001); no significant associations were observed for patient’s age, follow-up duration, physician sex, practice setting, hospital size, years in practice, thyroid case-mix, or the volumes of new or follow-up patients (all p > 0.05). In contrast, with ATA 2025 criteria (884 patients; 16 physicians), lower adequacy was associated with older age (aOR = 0.980 per year, 95% CI 0.969–0.992; p = 0.001), dose instability (aOR = 0.110, 95% CI 0.078–0.156; p < 0.001), female physician gender (vs male; aOR = 0.244, 95% CI 0.065–0.915; p = 0.037), and a higher volume of new patients (aOR = 0.967 per patient, 95% CI 0.943–0.991; p = 0.009). No significant associations were observed for follow-up duration, practice setting, hospital size, years in practice, thyroid case-mix, or follow-up patient volume (all p > 0.05).

## Discussion

In this multicenter cohort of low-risk DTC patients with excellent or indeterminate response, we found that the adequacy of levothyroxine therapy—defined against guideline-concordant TSH targets—depends not only on patient factors but also on physician- and service-level characteristics, and that these relationships shift materially when moving from ATA 2015 to ATA 2025. At the descriptive level, adequacy clustered with hospital size, years in practice, thyroid case-mix, and service volumes, and the pattern differed across the two guideline frameworks (**Table 2**). In the multilevel analysis, dose instability emerged as the most consistent predictor of inadequate control under both frameworks (aOR 0.183 under ATA 2015; aOR 0.110 under ATA 2025), while additional ATA 2025-specific signals included older age (lower odds), female physician sex (lower odds vs. male), and higher new-patient volume (lower odds). Concomitantly, the proportion adequately controlled increased when adequacy was defined with ATA 2025 reference-interval targets (from ~40% under ATA 2015 to ~68% under ATA 2025 in the regression samples), aligning with the intent of the 2025 update to simplify titration for excellent/indeterminate responders and reduce unnecessary suppression (3, 4).

In our opinion, these findings are timely and novel for three reasons. First, while prior real-world studies document suboptimal and heterogeneous attainment of target TSH and suggest that deviations often reflect prescribing patterns rather than assay variability (11), few analyses have quantified how clinician attributes and care-delivery features translate into adequacy after accounting for patient clustering—especially in the context of the ATA 2025 transition (6, 8, 11). Our results extend that line of evidence by showing that service structure and provider workload help explain residual variation once patient-level factors are controlled, and that the determinants of adequacy differ when targets are reference-interval–based rather than fixed numeric cutoffs. Second, by identifying dose instability as a high-magnitude, cross-framework correlate of inadequacy, we point to actionable levers (protocols to prevent dose oscillations, adherence and interaction checks, weight-change triggers, and titration pathways) that are likely to yield larger gains than further assay harmonization (17–20). Third, our results dovetail with the broader evidence base showing analytical concordance across modern TSH platforms and frequent over- or under-treatment in practice, thereby supporting the ATA 2025 emphasis on reference-range targets to enhance feasibility and mitigate cardiovascular and skeletal risks linked to prolonged suppression in low-risk survivors (6, 11, 21). In brief, these results align with the notion that identifying and correcting medication dose instability through targeted interventions is an effective strategy for optimizing treatment in patients with chronic diseases (22), as has been demonstrated in conditions such as hypertension (23), epilepsy (24), and neoplastic diseases (25).

Our findings align with and extend prior real-world reports showing suboptimal, heterogeneous attainment of guideline TSH targets in DTC, yet they add a physician- and service-level lens that has been largely missing. Multicenter series from Turkey (5) and Thailand (6) documented low “on-target” rates and pointed to practice inertia—particularly among more senior clinicians—as a key driver of over-suppression. Our previous multicenter study on low-risk DTC found low levothyroxine adequacy (26.0% at 12 months and 39.5% at last visit) and singled out dose instability as the principal determinant of long-term inadequacy, while discussing barriers to guideline adoption across institutions (8).

Our present work converges with these observations but goes further by quantifying, in multilevel models, how physician attributes (e.g., sex, workload) and hospital-level features relate to adequacy after adjusting for patient clustering, and by showing that these relationships shift materially when adequacy is judged with ATA 2025 reference-interval targets instead of ATA 2015 fixed cut-offs. An inverse association between female physician sex and treatment adequacy was observed under the ATA 2025 framework; however, this finding should be interpreted with caution. Given the observational design, this association does not imply causality and may reflect residual confounding or unmeasured structural factors. Although our models adjusted for key physician- and service-level variables, additional influences—such as workload distribution, consultation time, or differences in clinical practice organization—were not captured. In fact, some authors have documented that the physician’s sex can act as a marker of other behaviors that lead to different outcomes, rather than as an independent determinant. It is important not to assume that these are inherent and unchangeable. Instead, the drivers of these observed differences should be investigated to elucidate the positive behaviors that lead to outcomes (26).

Furthermore, our results dovetail with evidence that modern TSH assays are analytically concordant and that deviations from targets in practice arise primarily from implementation patterns rather than assay variability—supporting the ATA 2025 move toward reference-based goals for excellent/indeterminate responders (11). Importantly, the 2025 ATA update formalizes the DATA framework (diagnosis, risk/benefit assessment, treatment decisions, response assessment) and replaces fixed numeric cut-offs with assay-specific, reference-interval targets—”within normal reference range” for excellent/indeterminate responders and “below normal reference range” for biochemical/structural persistence—thereby aligning monitoring with analytical reality and intended de-escalation in survivorship. Beyond observational cohorts, contemporary evidence from a systematic review/meta-analysis and a multicenter cohort shows no clear survival benefit from sustained TSH suppression in intermediate/high-risk DTC, reinforcing the rationale for de-escalation in low-risk survivors and underscoring the potential harms of unnecessary suppression (7).

Discrepancies between our adequacy rates and those previously published likely reflect several factors. Firstly, it is necessary to consider the effects of the framework. When we re-classified adequacy using ATA 2025, the share of adequately controlled patients rose from 41.5% (364/878) to 68.8% (608/884) in the same regression cohorts, consistent with the 2025 guideline’s intent to de-escalate and broaden acceptable TSH ranges for excellent/indeterminate responders—whereas earlier cohorts were judged against narrow numeric bands (ATA 2015). Second, some prior reports pooled all risk strata or lacked hierarchical modeling, potentially conflating provider behavior with patient-level heterogeneity. By restricting to low-risk PTC with excellent/indeterminate response and modeling patients nested within physicians, we isolated dose instability as a cross-framework, high-magnitude correlate (aOR 0.183 under ATA 2015; 0.110 under ATA 2025) and identified provider/service levers (e.g., physician sex and new-patient volume under ATA 2025) that may be obscured in non-hierarchical designs. Third, our analysis has considered the changes induced by the adoption of the ATA 2025 criteria over the older ATA 2015 criteria, whereas many previous series reflected only the ATA 2015 era or mixed practices. This transition plausibly explains why our contrasts between frameworks are more pronounced. Beyond reconciling numbers, our study adds actionable novelty: it demonstrates, with prospectively relevant stratification, that adopting ATA 2025 reference-interval targets can substantially increase adequacy without relying on further assay harmonization, and it delineates concrete system-level targets—preventing dose instability and managing service load—to operationalize safe de-escalation in the population that constitutes the bulk of thyroid cancer survivorship.

Our findings have direct implications for clinical practice and healthcare organization. The consistent association between dose instability and inadequate TSH control identifies a modifiable target for quality improvement and highlights the need for proactive strategies to reduce levothyroxine dose variability. These may include standardized titration protocols, systematic assessment of factors affecting levothyroxine requirements (such as weight changes, adherence, and drug interactions), and scheduled reassessment of dosing needs (20, 27, 28). In addition, implementation of structured clinical pathways, audit-and-feedback programs, decision-support tools, and clinic-level monitoring of TSH target attainment may facilitate adoption of ATA 2025 reference-range targets and reduce unnecessary TSH suppression (29–31). Such measures are particularly relevant given the persistently low rates of target TSH attainment reported in real-world cohorts and the growing evidence that chronic TSH suppression in non-high-risk disease increases cardiovascular and skeletal risks without clear survival benefit (7, 32–35). Collectively, these strategies support the more individualized and risk-adapted management approach promoted by the ATA 2025 guidelines (20, 36).

Our study has strengths and weaknesses. This design leverages a large, multicenter real-world cohort of low-risk PTC patients with excellent/indeterminate response, enhancing generalizability across diverse providers and hospital settings, and applies two prespecified adequacy frameworks to the same patients, offering a timely head-to-head view of how the transition to reference-interval targets affects clinical performance metrics. Methodologically, the use of multilevel logistic regression with random intercepts appropriately accounts for patient clustering within physicians, while explicitly modeling patient-, physician-, and service-level covariates; furthermore, we quantify the impact of dose instability—a modifiable implementation lever—rather than inferring it indirectly.

Nevertheless, the retrospective design limits causal inference and is susceptible to residual confounding. The adoption of the ATA 2025 criteria among clinicians is still in a very early stage, so clinician behavior may still be evolving; and although laboratory testing followed routine certified methods, heterogeneity in local assays and reference intervals may introduce unavoidable noise in TSH categorization under ATA 2025. In addition, some exposure variables (e.g., physician workload, case-mix) were captured at the service level, which may mask within-physician temporal variation, and adherence or drug–drug interactions were not systematically evaluated. An additional limitation is the relatively small number of physicians included in the study, inherent to the multicenter design. Although multilevel modeling was used to account for clustering, the limited number of physician-level units may reduce statistical power to detect provider-level effects and may affect the generalizability of these findings. Consequently, associations involving physician characteristics should be interpreted cautiously and considered hypothesis-generating. Finally, while excluding biochemical/structural incomplete responders focuses the analysis on the most common and clinically relevant scenarios for de-escalation, it limits extrapolation to patients requiring intensive suppression. Overall, the design, scope, and modeling strategy provide robust, practice-informing signals, but confirmation in prospective, longitudinal studies with standardized titration protocols and harmonized laboratory reporting is warranted.

In summary, in this large, real-world cohort of low-risk PTC survivors with excellent or indeterminate response, we show that levothyroxine adequacy is not solely a patient-level outcome: it improves substantially when applying ATA 2025 reference-interval targets (vs. ATA 2015 fixed cut-offs) and remains strongly shaped by clinician- and service-level factors, with dose instability emerging as the most consistent, high-magnitude determinant of inadequate control. These findings operationalize the intent of ATA 2025—to simplify titration and avoid unnecessary suppression in low-risk survivors—and align with evidence that assay variability is minor relative to prescribing/implementation patterns, and that prolonged TSH suppression lacks clear survival benefit while increasing cardiovascular and skeletal risks.

Looking ahead, prospective, multicenter studies—embedding standardized de-escalation pathways, proactive prevention of dose instability (adherence checks, interaction screening, weight-change triggers), and harmonized laboratory reporting—are warranted to validate causal mechanisms and to test scalable implementation strategies across diverse systems. By embracing ATA 2025 reference-based targets and hard-wiring processes that stabilize dosing, endocrine services can raise on-target rates, reduce avoidable harm, and ultimately improve quality of life for the largest segment of the thyroid cancer population.

## Data Availability

The raw data supporting the conclusions of this article will be made available by the authors, without undue reservation.
