# Supplementary material for: Physician and hospital determinants of levothyroxine treatment adequacy in low-risk papillary thyroid carcinoma
Source: Front Endocrinol (Lausanne). 2026 Aug 4;17:1820537. doi: 10.3389/fendo.2026.1820537 (PMC13481202; doi:10.3389/fendo.2026.1820537)
Supplement: Supplementary file 1 [file Table1.docx]

**Physician and Hospital Determinants of Levothyroxine Treatment Adequacy in Low‑Risk Papillary Thyroid Carcinoma**

**SUPPLEMENTARY MATERIAL**

**Table S1**. Methods for measuring serum thyrotropin by the investigators participating in the study, indicating the reference interval of the assay used

| **Manufacturer** | **Instrument** | **Investigator (number, %)** | **Patients (number, %)** | **Reference interval (mcU/ml)** |
| --- | --- | --- | --- | --- |
| Abbott | Alinity | 3 (17.6) | 155 (15.3) | 0.35-4.94 |
| Beckman | DxI | 3 (17.6) | 257 (25.3) | 0.30-5.30 |
| Roche | Cobas | 2 (11.8) | 86 (8.5) | 0.25-5.00 |
|  | Elecsys | 2 (11.8) | 153 (15.1) | 0.30-4.20 |
| Siemens | Advia Centaur | 1 (5.9) | 61 (6.0) | 0.55-5.00 |
|  | Atellica | 6 (35.3) | 304 (29.9) | 0.55-4.78 |

Abbreviations: DxI; diagnostics immunoassay

**Table S2**. Comparison of patients’ characteristics in studied subjects with adequacy and inadequacy of levothyroxine treatment in the group of subjects with excellent or indeterminate response at the last follow-up visit according to the ATA 2015 and 2025 criteria

|  | **Adequacy of levothyroxine therapy in patients with excellent or indeterminate response** | | | | | |
| --- | --- | --- | --- | --- | --- | --- |
|  | **Evaluation using ATA 2015 criteria (n=954)** | | | **Evaluation using ATA 2025 criteria (n=963)** | | |
|  | **Adequate (n=383)** | **Inadequate (n=571)** | **P value** | **Adequate (n=650)** | **Inadequate (n=313)** | **P value** |
| **Age, years** | 48 (38-58) | 49 (39-59) | 0.238 | 49 (39-59) | 46 (37-59) | 0.043 |
| **Time of follow-up, months** | 93 (53-156) | 76 (44-132) | 0.002 | 88 (52-153) | 74 (40-125) | 0.001 |
| **Dose stability*** |  |  | <0.001 |  |  | <0.001 |
| **Stable** | 310 (85.2) | 267 (51.9) |  | 490 (80.6) | 92 (33.3) |  |
| **Unstable** | 54 (14.8) | 247 (48.1) |  | 118 (19.4) | 184 (66.7) |  |

*Using the ATA 2015 criteria, the dose stability could only be evaluated in 878 patients (364 with adequate control and 514 with inadequate control). Using the ATA 2025 criteria, this variable was evaluated in 884 patients (608 with adequate control and 276 with inadequate control).

Abbreviations: ATA, American Thyroid Association
